# Is adult-onset separation anxiety disorder a trauma-stress-related disorder? A preliminary report

**DOI:** 10.1017/S1092852925000239

**Published:** 2025-07-21

**Authors:** Camilla Gesi, Annalisa Cordone, Claudia Carmassi, Liliana Dell’Osso

**Affiliations:** 1 Department of Psychiatry and Addiction, ASST Fatebenefratelli-Sacco, Milan, Italy; 2 University of Milan, Milan, Italy; 3 Department of Clinical and Experimental Medicine, University of Pisa, Pisa, Italy

**Keywords:** Separation anxiety disorder, adult onset, adult separation anxiety, trauma, trauma and stress-related disorders

## Abstract

**Background:**

The DSM-5 recognized that the separation anxiety disorder (SEPAD) may span the entire life course or have an adult-onset. Epidemiological data indicated a 23%–69% prevalence of SEPAD in clinical settings and a high comorbidity with both prolonged grief disorder (PGD) and post-traumatic stress disorder (PTSD). Some authors hypothesize that while life threat represents the key trigger of PTSD, disruptions or threats to interpersonal bonds lead to PGD and SEPAD. This study aims to test the hypothesis that adult-onset SEPAD might be a trauma-related disorder, triggered by events threatening to interpersonal bonds.

**Methods:**

The sample included 106 consecutive adult outpatients with anxiety and/or mood disorders. SEPAD was diagnosed according to DSM-5 criteria by means of the Structured Clinical Interview for Separation Anxiety Symptoms (SCI-SAS). The Adult Separation Anxiety Checklist (ASA-27) was used to assess symptoms severity. To assess exposure to trauma, the SCID-5 criterion A form for PTSD was administered. Traumatic events were coded as directly experienced (self) or involving close ones (others). Lifetime exposure to separation events was also assessed.

**Results:**

60.4% of participants were categorized as not having SEPAD in adulthood or in childhood (NO-SEPAD), 18.9% as childhood-onset SEPAD, and 20.8% as adult-onset SEPAD. Controlling for comorbid disorders, lifetime traumatic events involving self and separation events, traumatic events involving others significantly predicted adult-onset SEPAD. A significant correlation between the age at trauma exposure and the age of SEPAD onset was found.

**Conclusions:**

Our results are consistent with the hypothesis that adult-onset SEPAD may represent an event-related disorder.

## Introduction

Separation anxiety disorder (SEPAD) is the most frequent manifestation of anxiety during childhood, and it was for a long time considered a time-limited condition, vanishingly rare among adolescents and adults.^1^ Besides a large amount of data linking juvenile SEPAD to the heightened risk of developing panic disorder and other mental disorders during adulthood^2^^–^^7^ in the last decades growing attention has been focusing on the evidence that SEPAD may continue as such through the adult life and to the possibility that it may even begin at any age.^8^^–^^13^ Based on this literature, the fifth edition of the Diagnostic and Statistical Manual of Mental Disorders (DSM-5) has removed the age restriction on the diagnosis of SEPAD and has assigned the category to the section of anxiety disorders,^14^ effectively recognizing that the disorder may span the entire life course.

As described in DSM-5, individuals with SEPAD experience excessive distress when separation from home or major attachment figures is anticipated or occurs; they worry about the well-being or potential death of their loved ones, particularly when separated from them; and they are preoccupied with knowing the whereabouts of their attachment figures, feeling compelled to stay in touch with them.

A few epidemiological studies evaluated the prevalence of SEPAD in the general population. Findings from the National Comorbidity Survey (NCS-R), involving the English-speaking household US population, have indicated that SEPAD has a lifetime prevalence in the adult general population of 6.6%.^15^ More recently, a WHO epidemiological survey conducted across 18 countries worldwide^16^ showed an overall lifetime prevalence of SEPAD as high as 4.8%, with large variability across different countries. SEPAD has also been shown to be highly frequent in several clinical settings, with prevalence estimates ranging from 23% to about 69%, depending on the nature of the sample.^8^^,^^9^^,^^10^^,^^12^^,^^17^^–^^21^ A particularly high level of comorbidity has been shown between SEPAD and both prolonged grief disorder (PGD) and post-traumatic stress disorder (PTSD).^21^^,^^22^ In a study conducted among pregnant women in war-affected Timor-Leste, women with core SEPAD symptoms reported exposure to multiple traumatic losses and intimate partner violence and showed a pattern of comorbidity with PTSD, which differentiated them from women with low or limited SEPAD symptoms.^23^ Further data from West Papua refugees^24^ highlighted the mediating role of SEPAD symptoms in the relationship between traumatic losses and worry about family and PTSD symptoms. A study conducted on Bosnian Refugees^25^ found that virtually all participants with adult SEPAD had PTSD, although the majority of those with PTSD did not have adult SEPAD. Interestingly, authors proposed that traumas might have distinctive effects, with life threat being the key trigger of PTSD and disruptions or threats to interpersonal bonds leading to grief and SEPAD. Enriching such a perspective, we propose that SEPAD beginning during adulthood might ultimately be conceptualized as a trauma-related disorder, differentiating as such from forms beginning during childhood and either recovering before or continuing throughout adulthood. The present study aims to test the hypothesis that adult-onset SEPAD is associated with experiences of intense personal insecurity and/or worry about family, especially focusing on both traumatic and separation events that may be perceived as threatening interpersonal bonds.

## Methods

### Sample

Subjects with a principal diagnosis of mood (either depressive or bipolar) or anxiety disorder confirmed by the structured clinical interview for DSM-5 (SCID-5) and giving consent to take part in the study, were consecutively enrolled at the adult psychiatric outpatient clinic of Pisa between April 2016 and February 2017. Patients with psychotic spectrum symptoms, or intellectual disability/cognitive impairment before the index assessment were excluded. Subjects reporting substance use in the previous 3 years or endorsing DSM-5 criteria for substance use disorder during the lifetime were excluded as well. In a single session, participants were administered both self-report questionnaires and clinical interviews by experienced residents in psychiatry. The study was carried out in accordance with the Declaration of Helsinki and study design was reviewed by the University of Pisa Ethical Committee. All subjects were informed of the nature of study procedures and provided written informed consent prior to participation.

### Assessments

#### Separation anxiety

The Structured Clinical Interview for Separation Anxiety Symptoms (SCI-SAS) was administered to assess the presence of SEPAD. This interview evaluates each of the eight DSM-5 criterion symptoms of separation anxiety, separately for Childhood (SCI-SAS-C) and Adult symptoms (SCI-SAS-A). In proceeding with the DSM-5 guidelines, endorsement of three or more of the eight criterion symptoms [symptoms rated as 2 (often)] was used as a threshold to determine categorical (yes /no) diagnosis of SEPAD in childhood and adulthood. In addition, criterion B (ie duration of at least 4 weeks) and C (ie the disturbance causes clinically significant distress or impairment in social, academic and occupational, or other important areas of functioning) were required. The Adult Separation Anxiety Checklist (ASA-27)^26^ was used to assess the severity of symptoms of SEPAD. It consists of 27 items rated on a scale from 0 (this never happens) to 3 (this happens all the time), with a total score ranging from 0 to 81.

#### Traumatic and separation events

To assess trauma exposure, participants were administered the SCID-5 criterion A module for PTSD. Endorsement of this criterion was differently coded if the traumatic event was directly experienced (self) or involved close ones (others). Lifetime exposure to separation events included the following instances: break-up with a partner or a friend, separation/divorce from partner, death of a loved one, moving to another town.

### Statistical analyses

The mean values (±SD) of continuous variables such as age, and scale scores were compared between subject groups using the T test. Comparisons of categorical variables between groups were conducted using the chi-square test. Separation and traumatic events as predictors of adult-onset SEPAD (dependent variable) were investigated by a binary logistic regression analysis using sex and the diagnosis of panic, depressive and bipolar disorder as covariates. Data were analyzed using SPSS software version 24.0 (IBM Corp). All *p* values are 2-sided, and the statistical significance was set at *p* < .05.

## Results

Three hundred and sixty-seven subjects referring to the outpatient service were screened during the enrollment period. Among them, 89 (35.8%) were diagnosed with a psychotic spectrum disorder, 39 (15.7%) with cognitive impairment, 36 (14.6%) with intellectual disability, and 84 (33.9%) with substance use (58 current substance use, and 26 substance use disorder in their lifetime). Thus, 119 subjects (32.4%) subjects met the inclusion criteria and were proposed for participation in the study. Of these, 13 (10.9%) did not want to participate (6 because they did not have time; 4 because they were not interested; 3 because they did not want to sign the consent form). The final sample thus included a group of 106 consecutive adult psychiatric outpatients with anxiety and/or mood (either depressive or bipolar) disorders as a principal diagnosis. As to mood disorders, 27 patients had a diagnosis of major depression and 56 of bipolar disorder. Overall, 72 had an anxiety disorder (33 panic disorder, 39 other anxiety disorders).

Of the total cohort of 106 patients with mood and anxiety disorders, 64 (60.4%) were categorized as not having separation anxiety in adulthood or in childhood (NO-SEPAD), 20 (18.9%) with childhood-onset SEPAD, and 22 (20.8%) with adult-onset SEPAD.

Mean age of onset among adult-onset SEPAD was 39.4 ± 16.6 years. Main socio-demographic and clinical characteristics of the study sample are shown in Table 1. Dichotomous lifetime exposure to traumatic events involving others (but not involving self) was higher among subjects with adult-onset SEPAD (*p* = .017). The number of lifetime separation events did not differ among the three groups (Table 1). Controlling for sex, major depression, bipolar disorder, and panic disorder, as well as for lifetime traumatic events involving self and separation events, traumatic events involving others significantly predicted adult-onset SEPAD (*p* = .017) (Table 2).

**Table 1.** Socio-demographic and Clinical Characteristics of Study Sample

	No SEPAD *n* = 64	Childhood-onset SEPAD *n* = 20	Adult-onset SEPAD *n* = 22	Chi	Overall adult SEPAD	Chi
Sex						
Male Female	14 (21.9) 50 (78.1)	4 (20.0) 16 (80.0)	6 (27.3) 16 (72.7)	.371	10 (23.8) 32 (76.2)	.816
Marital status						
Never married Married/cohabiting Sep./div./widowed	26 (40.6) 21 (32.8) 17 (26.6)	8 (40.0) 9 (45.0) 3 (15.0)	6 (27.3) 11 (50.0) 5 (22.7)	3.184	14 (33.3) 20 (47.6) 8 (19.0)	.301
Employment status						
Unemployed Employed Other (student, housewife, retired)	21 (32.8) 20 (31.3) 23 (35.9)	3 (15.0) 11 (55.0) 6 (30.0)	6 (27.3) 10 (45.5) 6 (27.3)	4.711	9 (21.4) 21 (52.4) 12 (28.6)	.144
Education						
Up to 8 y 9–13 y 14 y or above	24 (37.5) 30 (46.9) 10 (15.6)	6 (30.0) 13 (65.0) 1 (5.0)	11 (50.0) 9 (40.9) 2 (9.1)	4.168	17 (40.5) 22 (52.4) 3 (7.1)	.427
Living status						
With parents With partner/child Alone	22 (34.4) 28 (43.8) 14 (21.9)	6 (30.0) 10 (50.0) 4 (20.0)	2 (9.1) 15 (68.2) 5 (22.7)	5.716	8 (19.0) 25 (59.5) 9 (21.5)	.186
Depressive disorders	16 (25.0)	5 (25.0)	6 (27.3)	.047	11 (26.2)	.019
Bipolar disorders	33 (51.6)	10 (50.0)	13 (59.1)	.452	23 (54.8)	.104
Panic disorder	15 (23.6)	9 (33.3)	9 (33.3)	1.26	18 (42.9)	4.461*
Other anxiety	24 (23.4)	5 (25.0)	10 (45.5)	4.044	15 (35.7)	.003
Other disorders	6 (10.9)	3 (11.1)	2 (7.4)	3.38	5 (11.9)	.175
Exposure to traumatic events (self)	15 (23.4)	7 (35.0)	6 (27.3)	1.059	13 (31.0)	.737
Exposure to traumatic events (others)	2 (3.1)	2 (10.0)	5 (22.7)	8.169*	7 (16.7)	5.985*
Lifetime separation events						
None One Two or more	23 (35.9) 26 (40.6) 15 (23.4)	7 (35.0) 11 (55.0) 2 (10.0)	7 (31.8) 11 (50.0) 4 (18.2)	2.337	14 (33.3) 22 (52.4) 6 (14.3)	1.895
				**F**		
Age	45.2 (14.6)	46.6 (17.6)	50.6 (13.0)	1.060	48.7 (15.2)	−1.188
ASA–27 tot	25.5 (13.7)	32.7 (11.7)	32.4 (7.6)	4.129*	32.5 (9.5)	−2.885*

*Note:* Overall Adult adult SEPAD = all subjects with adult SEPAD, being childhood-onset SEPAD and adulthood-onset SEPAD together.

* *p*<.05.

**Table 2.** Binary Logistic Regression of Predictors of Adult-onset SEPAD

Coefficients^a^				
Model	Unstandardized coefficients	Standardized coefficients	wald	Sig.
	B (SE)	Beta		
Constant	−2.724 (.792)	.066	11.829	**.001**
Sex	.262 (.643)	1.299	.166	.684
Depressive disorder	.236 (0.768)	1.266	.095	.758
Bipolar disorder	1.146 (.694)	3.145	2.726	.099
Panic disorder	1.058 (.561)	2.880	3.557	.059
Separation events [none]			.280	.869
One Two or more	.013 (.590) .352 (.732)	1.013 1.422	.000 .231	.898 .630
Traumatic events (self)	.004 (.638)	1.004	.001	.995
Traumatic events (others)	2.606 (.990)	13.543	6.924	**.009**

Hosmer-Lemeshow test: χ^2^(8) = 3.263, p = .917.

Cox R^2^ = .103, Nagelkerke R^2^ = .159.

Percentage of overall correct prediction = 80.2%.

a Dependent variable: Diagnosis of adult-onset SEPAD.

**Figure 1.**
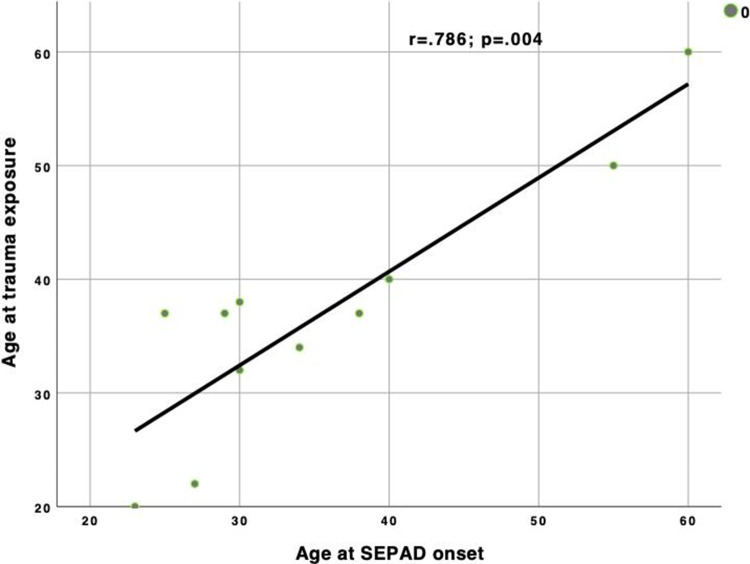
Correlation between age at trauma exposure (*other*) and age at SEPAD onset in the adult-onset SEPAD group.

Among subjects with adult-onset SEPAD with exposure to trauma of others, the age of onset significantly correlated with the age at trauma exposure (*r* = .786; *p* = .004) (Figure 1).

## Discussion

The present paper was designed to evaluate whether SEPAD arising during adulthood, without a history of childhood separation anxiety, might relate to separation or traumatic events. To this aim, we investigated both separation and traumatic events occurring during the lifespan in a clinical group of subjects with mood and anxiety disorders additionally undergoing assessment for SEPAD. We found that subjects with adult-onset SEPAD show a positive history for traumatic events to a significantly greater extent than subjects with child-onset SEPAD or with no SEPAD. Moreover, traumatic events occurring to closest others significantly predicted adult-onset (but not childhood-onset) SEPAD, while separation events and traumas occurring to oneself did not. Intriguingly, participants with adult-onset SEPAD showed a positive, significant correlation between age at trauma exposure and the age of SEPAD onset.

Our results are consistent with the hypothesis that SEPAD symptoms manifesting for first-time during adulthood may represent an event-related disorder, having distinctive features from forms beginning during the developmental period and either recovering by adulthood or continuing as such through the adult life. This hypothesis was previously suggested by Silove et al.^25^ who found a 100% prevalence of SEPAD among Bosnian refugees with PTSD and speculatively called the role of events threatening relationship bonds in the pathophysiology of adult SEPAD. Furthering Silove’s findings, our data confirm the relationship between traumatic events and SEPAD, also distinguishing between subjects in whom SEPAD is a lifelong disorder from those who develop SEPAD during adulthood, with no history of childhood SEPAD.

In addition, we found that only traumatic events involving close attachment figures play a significant role in predicting adult-onset SEPAD. On the contrary, traumas concerning personal safety and separation events do not. This is consistent with most core symptoms of SEPAD, involving fear that harm could befall to significant others and the need to maintain proximity to them (DSM-5), and aligns with previously proposed models putting forward that traumas involving life threat are the key trigger of PTSD, and disruptions or threats to interpersonal bonds rather result in grief and SEPAD (ADAPT, adaptation and development after persecution and trauma).^26^ Our results are also consistent with negative results about the role of actual separation experiences in determining childhood SEPAD and confirm the relative weight of environmental vs genetic factors from childhood to adulthood in the pathophysiology of anxiety.^27^^,^^28^ As tailored treatments are available for trauma-related disorders, this may ultimately have important implications for differentiating treatment choices on the basis of age of first onset of separation anxiety symptoms.

For example, psychotherapeutic treatment of adult-onset SEPAD could profitably incorporate techniques derived from trauma-focused psychotherapies, which are the current mainstay treatment for PTSD, with larger effect sizes than other currently available treatments. It is noteworthy, in this regard, that eye movement desensitization and reprocessing (EMDR), trauma-focused cognitive behavioral therapy (TF-CBT), and exposure techniques have already been tested in cases of childhood SEPAD following traumatic separation. Our results are still to be viewed considering some limitations. First, the sample size is quite small, dulling the generalizability of the findings and highlighting the need for replication studies in larger samples. Second, the study lacks a longitudinal perspective, and both traumatic/separation events and SEPAD symptom assessment relied on participants’ recall. Third, due to the low caseness of traumatic events, we could not investigate the impact of multiple traumas in the development of SEPAD, which was instead done for separation events. Fourth, we did not consider whether positive life events (ie marriage, childbirth, movings) might play a role in SEPAD onset. Furthermore, we must acknowledge that the prevalence of adult SEPAD was quite high in our sample (41.5%). Despite aligning with rates from some previous studies, we cannot rule out that such a high prevalence is due to the overlap between DSM-5 diagnostic criteria of SEPAD and some symptoms of PTSD, especially those in the domain of avoidance. In this regard, our finding of traumatic events involving close attachment figures as a key trigger of adult SEPAD may also help to disentangle the two psychopathological dimensions. Finally, the exclusion of subjects with current substance use/past substance use disorder, while ruling out a potential confounding effect of substance use, hampers the generalizability of results to dual diagnosis populations. Yet, our study contributes to inquiring about our knowledge on separation anxiety and may be of preliminary ground for further research on adult-onset SEPAD.

## References

[1] Copeland WE, Angold A, Shanahan L, et al. Longitudinal patterns of anxiety from childhood to adulthood: the Great Smoky Mountains Study. J Am Acad Child Adolesc Psychiatry. 2014;53(1):21–33. 24342383 10.1016/j.jaac.2013.09.017 PMC3939681

[2] Kossowsky J, Pfaltz MC, Schneider S, et al. The separation anxiety hypothesis of panic disorder revisited: a meta-analysis. Am J Psychiatry. 2013;170:768–781. 23680783 10.1176/appi.ajp.2012.12070893

[3] Silove D, Rees S. Separation anxiety disorder across the lifespan: DSM-5 lifts age restriction on diagnosis. Asian J Psychiatr. 2014;11:98–101. 25453710 10.1016/j.ajp.2014.06.021

[4] Silove D, Momartin S, Marnane C, Steel Z, Manicavasagar V. Adult separation anxiety disorder among war-affected Bosnian refugees: comorbidity with PTSD and associations with dimensions of trauma. J Trauma Stress. 2010;23(1):169–172. 20135680 10.1002/jts.20490

[5] Battaglia M, Pesenti-Gritti P, Medland SE, Ogliari A, Tambs K, Spatola CAM. A genetically informed study of the association between childhood separation anxiety, sensitivity to CO2, panic disorder, and the effect of childhood parental loss. Arch Gen Psychiat. 2009;66(1):64–71. 19124689 10.1001/archgenpsychiatry.2008.513

[6] Lipsitz JD, Martin LY, Mannuzza S, et al. Childhood separation anxiety disorder in patients with adult anxiety disorders. Am J Psychiatry. 1994;151(6):927–929. 8185008 10.1176/ajp.151.6.927

[7] Vanderwerken LC, Jacobs SC, Parkes CM, Prigerson HG. An exploration of associations between separation anxiety in childhood and complicated grief in later life. J Ner Ment Dis. 2006;194(2):121–123. 10.1097/01.nmd.0000198146.28182.d5 16477190

[8] Manicavasagar V, Silove D. Is there an adult form of separation anxiety disorder? A brief clinical report. Aust N Z J Psychiatry. 1997;31(2):299–303. 9140640 10.3109/00048679709073835

[9] Manicavasagar V, Silove D, Curtis J. Separation anxiety in adulthood: A phenomenological investigation. Compr Psychiatry. 1997;38(5):274–282. 9298320 10.1016/s0010-440x(97)90060-2

[10] Manicavasagar V, Silove D, Curtis J, Wagner R. Continuities of separation anxiety from early life into adulthood. J Anxiety Disord. 2000;14(1):1–18. 10770232 10.1016/s0887-6185(99)00029-8

[11] Manicavasagar V, Silove D, Rapee R, Waters F, Momartin S. Parent–child concordance for separation anxiety: A clinical study. J Affect Disord. 2001;65(1):81–84. 11426514 10.1016/s0165-0327(00)00241-x

[12] Pini S, Gesi C, Abelli M, et al. The relationship between adult separation anxiety disorder and complicated grief in a cohort of 454 outpatients with mood and anxiety disorders. J Affect Disord. 2012;143(1–3):64–68. doi:10.1016/j.jad.2012.05.026. 22832169

[13] Silove D, Slade T, Marnane C, Wagner R, Brooks R, Manicavasagar V. Separation anxiety in adulthood: Dimensional or categorical? Compr Psychiatry. 2007;48(6):546–553. 17954140 10.1016/j.comppsych.2007.05.011

[14] Silove D, Rees S. Separation anxiety disorder across the lifespan: DSM-5 lifts age restriction on diagnosis. Asian J Psychiatr. 2014;11:98–101. 25453710 10.1016/j.ajp.2014.06.021

[15] Shear K, Jin R, Ruscio AM, Walters EE, Kessler RC. Prevalence and correlates of estimated DSM-IV child and adult separation anxiety disorder in the National Comorbidity Survey Replication. Am J Psychiatry. 2006;163(6):1074–1083. 16741209 10.1176/appi.ajp.163.6.1074 PMC1924723

[16] Silove D, Alonso J, Bromet E, Gruber M, Sampson N, Scott K. Pediatric-onset and adult-onset separation anxiety disorder across countries in the world mental health survey. Am J Psychiatry. 2015;172(7):647–656. 26046337 10.1176/appi.ajp.2015.14091185 PMC5116912

[17] Carmassi C, Gesi C, Corsi M, et al. Adult separation anxiety differentiates patients with complicated grief and/or major depression and is related to lifetime mood spectrum symptoms. Compr Psychiatry. 2015;58:45–49. 25595519 10.1016/j.comppsych.2014.11.012

[18] Gesi C, Abelli M, Cardini A, et al. Separation anxiety disorder from the perspective of DSM-5: clinical investigation among subjects with panic disorder and associations with mood disorders spectrum. CNS Spectr. 2016;21(1):70–75. 25704393 10.1017/S1092852914000807

[19] Dell’Osso L, Carmassi C, Musetti L, et al. Lifetime mood symptoms and adult separation anxiety in patients with complicated grief and/or post-traumatic stress disorder: a preliminary report. Psychiatry Res. 2012;198(3):436–440. 22436352 10.1016/j.psychres.2011.12.020

[20] Wijeratne C, Manicavasagar V. Separation anxiety in the elderly. J Anxiety Disord. 2003;17(6):695–702. 14624819 10.1016/s0887-6185(02)00239-6

[21] Carmassi C, Gesi C, Corsi M, et al. Adult separation anxiety differentiates patients with complicated grief and/or major depression and is related to lifetime mood spectrum symptoms. Compr Psychiatry. 2015;58:45–49. 25595519 10.1016/j.comppsych.2014.11.012

[22] Gesi C, Carmassi C, Shear KM, et al. Adult separation anxiety disorder in complicated grief: an exploratory study on frequency and correlates. Compr Psychiatry. 2017;72:6–12. 27683967 10.1016/j.comppsych.2016.09.002

[23] - Silove DM, Tay AK, Tol WA, et al. Patterns of separation anxiety symptoms amongst pregnant women in conflict-affected Timor-Leste: associations with traumatic loss, family conflict, and intimate partner violence. J Affect Disord. 2016;205:292–300. 27552593 10.1016/j.jad.2016.07.052

[24] Tay AK, Rees S, Chen J, Kareth M, Silove D. Pathways involving traumatic losses, worry about family, adult separation anxiety and posttraumatic stress symptoms amongst refugees from West Papua. J Anxiety Disord. 2015;35:1–8. 26275507 10.1016/j.janxdis.2015.07.001

[25] Silove D, Momartin S, Marnane C, Steel Z, Manicavasagar V. Adult separation anxiety disorder among war-affected Bosnian refugees: comorbidity with PTSD and associations with dimensions of trauma. J Trauma Stress. 2010;23(1):169–172. 20135680 10.1002/jts.20490

[26] - Silove D, & Steel Z. Understanding community psychosocial needs after disasters: Implications for mental health services. J Postgrad Med. 2006;52(2):121–125. 16679676

[27] Costa B, Pini S, Martini C, et al. Mutation analysis of oxytocin gene in individuals with adult separation anxiety. Psychiatry Res. 2009;168(2):87–93. 19473710 10.1016/j.psychres.2008.04.009

[28] Waszczuk MA, Zavos HM, Gregory AM, Eley TC. The phenotypic and genetic structure of depression and anxiety disorder symptoms in childhood, adolescence, and young adulthood. JAMA Psychiatry. 2014;71(8):905–916. 24920372 10.1001/jamapsychiatry.2014.655

